# *Vitisshizishanensis*, a new species of the grape genus from Hubei province, China

**DOI:** 10.3897/phytokeys.184.70045

**Published:** 2021-11-02

**Authors:** Zhi-Yao Ma, Jun Wen, Qiang Fu, Xiu-Qun Liu

**Affiliations:** 1 Key Laboratory of Horticultural Plant Biology (Ministry of Education), College of Horticulture and Forestry Science, Huazhong Agricultural University, Wuhan 430070, China National Museum of Natural History Washington D.C. United States of America; 2 Department of Botany, National Museum of Natural History, MRC166, Smithsonian Institution, Washington, D.C. 20013-7012, USA Huazhong Agricultural University Wuhan China

**Keywords:** Grape, phylogenomics, taxonomy, Vitaceae, *Vitis*

## Abstract

*Vitisshizishanensis* (Vitaceae), a new species from Hubei, China, is described and illustrated. It is morphologically similar to *V.flexuosa* and *V.bryoniifolia*, but differs in leaf lobing and pubescence. It can be easily distinguished from the two species based on its glabrous or with very sparse arachnoid tomentum on the abaxial mature leaf surface, and its unlobed to 3–7 lobed leaves. A detailed description, along with photographs for the new species, and a table for morphological comparisons with similar *Vitis* species, are also provided.

## Introduction

**Figure 1.**
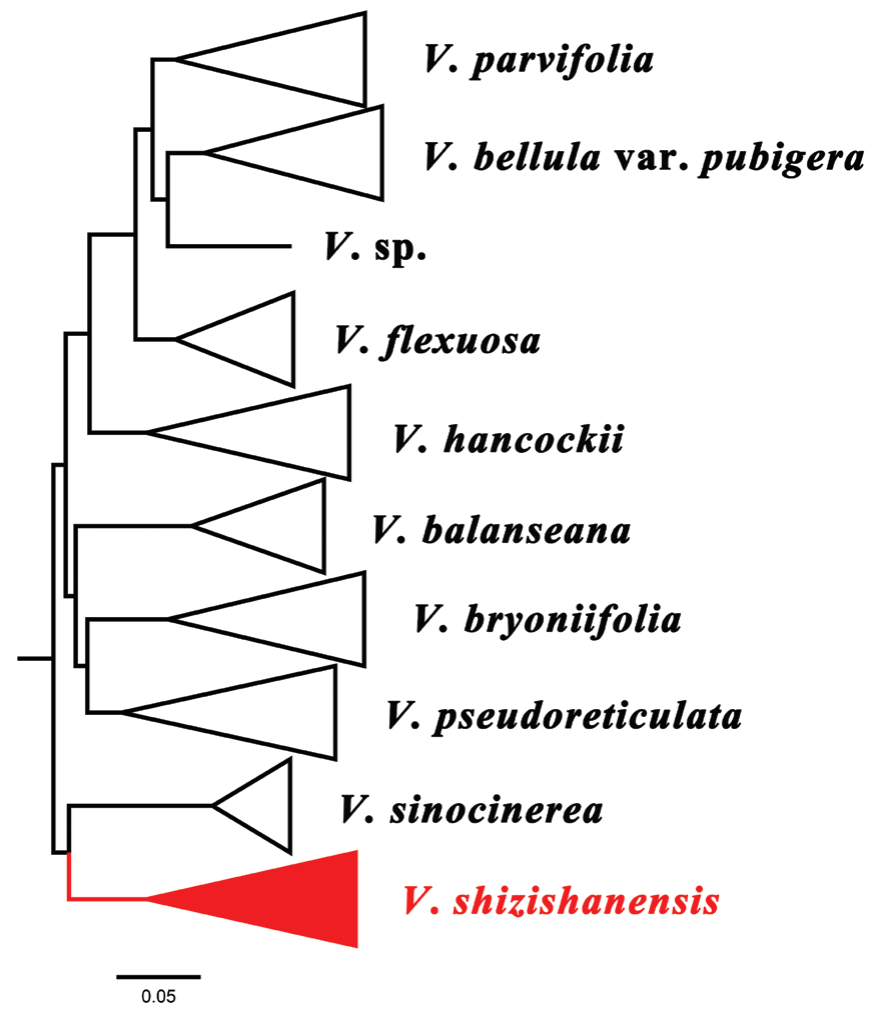
Simplified phylogenetic relationships of the *V.bryoniifolia* clade based on Ma et al. 2020.

The grapes (*Vitisvinifera* L.) represent one of the earliest domesticated and the most widely cultivated economic fruits in the world, as the source for grapes, raisins, and wine (Myles et al. 2011; Gerrath et al. 2015; Wen et al. 2018b). The grape genus *Vitis* L. contains ca. 70 species with an intercontinental disjunct distribution in North America (to northern South America), East Asia and Europe to West Asia (Galet 1988; Chen et al. 2007; Moore and Wen 2016; Wen et al. 2018a, 2018b). There are ca. 40 native species of *Vitis* in East Asia and most of them occur in China (Chen et al. 2007; Wan et al. 2008). Based on recent studies on molecular phylogeny and morphology of *Vitis*, a robust phylogenetic framework of *Vitis* has been reconstructed (Tröndle et al. 2010; Péros et al. 2011; Zecca et al. 2012; Aradhya et al. 2013; Wan et al. 2013; Liu et al. 2016; Ma et al. 2018a). However, due to rapid evolutionary radiations and extensive reticulate evolution of *Vitis* (Aradhya et al. 2013; Wan et al. 2013; Ma et al. 2018a, 2018b; Wen et al. 2018a), the species delimitation of *Vitis* is still controversial and the number of species of *Vitis* needs to be further assessed (Chen et al. 2007; Wan et al. 2008; Wen et al. 2018b; Ma et al. 2016, 2018b, 2020). Taxonomic challenges of some *Vitis* species are caused by their morphological similarity and overlapping geographic distribution (Chen et al. 2007; Moore and Wen 2016). A very complex group of *Vitis* is the *V.bryoniifolia* clade and its close allies (Ma et al. 2020). The phylogenetic relationships of the *V.bryoniifolia* clade have been reconstructed recently with robust support, which indicated that some samples previously difficult to identify need to be treated as a different species distinct from *V.bryoniifolia* based on molecular phylogenetic evidence (Ma et al. 2020) (Fig. 1). The leaf shape of this species shows a high level of phenotypic plasticity, varying from unlobed to 3–7 lobed, which caused problems for species identifications (Ma et al. 2020). After consulting relevant literature (Li et al. 1996; Wang et al. 2000; Chen et al. 2007; Wan et al. 2008) and our extensive field studies in East Asia, we herein propose to describe the new species, *Vitisshizishanensis* Z.Y.Ma, J. Wen, Q. Fu & X-Q. Liu.

## Material and methods

Descriptions and measurements of morphological characters of the new species were based on field observations of living plants at the type locality and specimens in the herbarium of Huazhong Agricultural University (CCAU) and the United States National Herbarium (US). We also examined herbarium specimens of *Vitis* comparatively from the following herbaria: CCNU, CSFI, HIB, HNNU, HUNST, HZU, JIU, JXCM, NYA, PE, and WH (abbreviations following Thiers 2020), and from images of type specimens and dried herbarium specimens on the Chinese Virtual Herbarium Website (http://www.cvh.ac.cn/), JSTOR Global Plants (http://plants.jstor.org), National Specimen Information Infrastructure (http://www.nsii.org.cn/), and Sharing Platform of IBK (http://www.gxib.cn/spIBK/).

## Taxonomic treatment

### Vitis shizishanensis

Taxon classificationPlantaeVitalesVitaceae

Z.Y.Ma, J.Wen, Q.Fu & X.Q.Liu sp. nov.

0A6A496C-22B5-5DCE-A353-35D34F09E80E

urn:lsid:ipni.org:names:77221513-1

**Figure 2.**
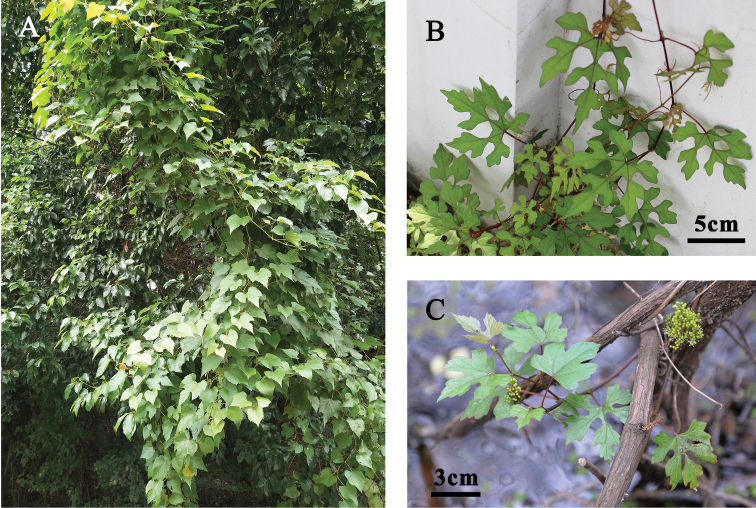
*Vitisshizishanensis* Z.Y.Ma, J.Wen, Q.Fu & X.Q.Liu, sp. nov. **A** habit **B** individual with 5–7 deeply lobed leaves **C** a flowering branch with 3–5 deeply lobed leaves.

**Figure 3.**
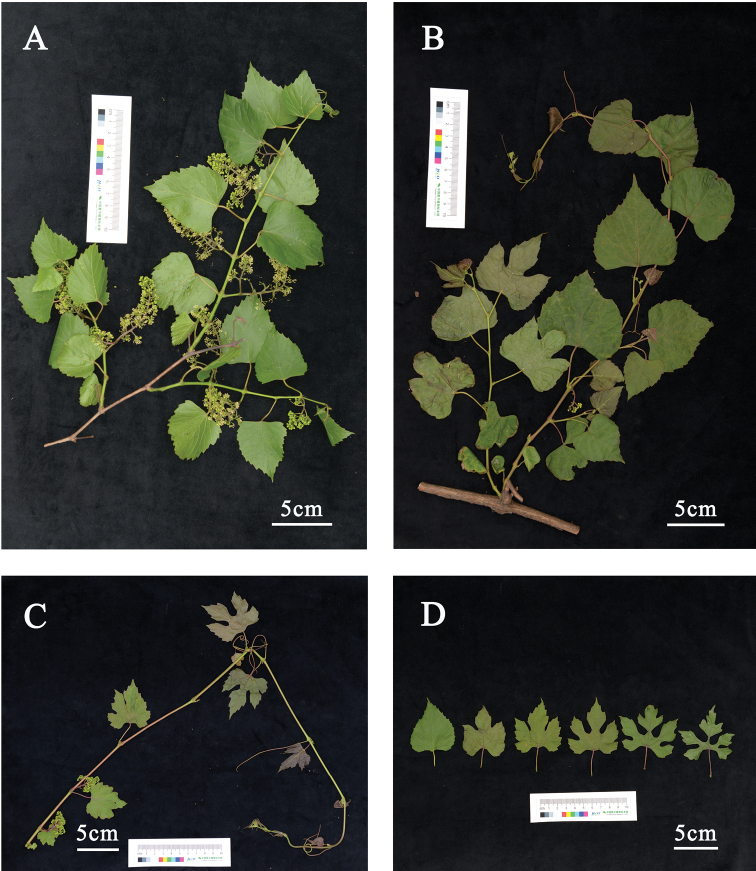
Branches and leaves of *Vitisshizishanensis* sp. nov. (*X.Q. LIU 755*) **A** branches with unlobed leaves **B** branches with unlobed to 3 lobed leaves **C** branches with 3–5 deeply lobed leaves **D** unlobed to 5–7 deeply lobed leaves.

**Figure 4.**
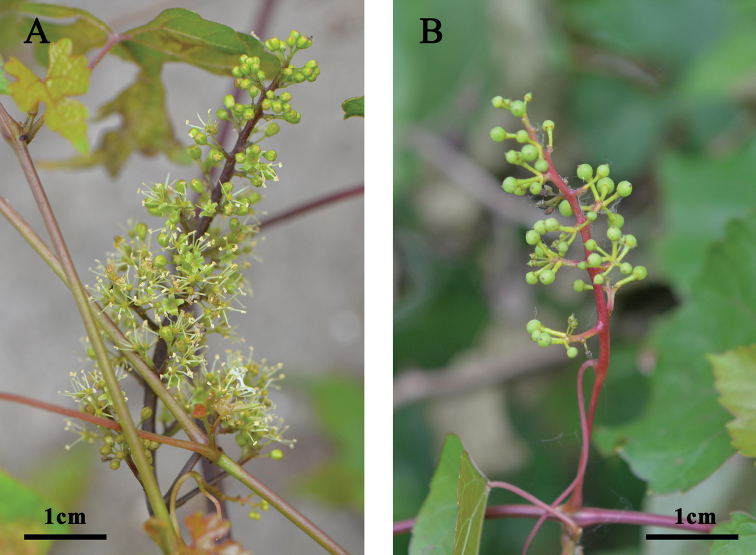
Inflorescences of *Vitisshizishanensis* sp. nov. **A** male flowers **B** female flowers after anthesis.

**Figure 5.**
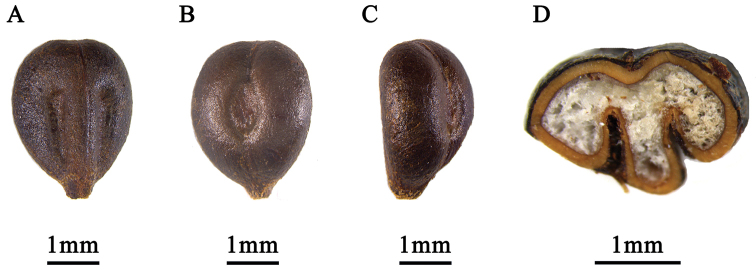
Seeds of *Vitisshizishanensis* sp. nov. **A** ventral view **B** dorsal view **C** lateral view **D** transverse section.

**Figure 6.**
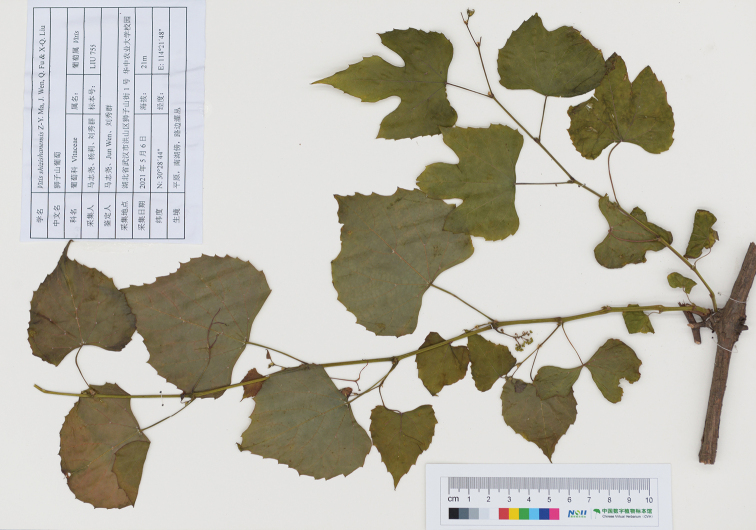
Holotype of *Vitisshizishanensis* sp. nov. Z.Y.Ma, J. Wen, Q. Fu & X-Q. Liu (*X.Q. LIU 755*).

Figures 2 , 3 , 4 , 5 , 6

#### Type.

China. Hubei: Wuhan City, Shizishan Mountain, 30°28'44"N, 114°21'48"E, 21 m, 6 May 2021, in fl., *X.Q. LIU 755* (holotype: CCAU!; isotypes: CCAU!, US!).

#### Diagnosis.

*Vitisshizishanensis* is morphologically similar to *V.bryoniifolia* Bunge, *V.flexuosa* Thunb, *V.sinocinerea* W. T. Wang, and *V.bellula* (Rehder) W. T. Wang, but differs from the *V.bryoniifolia*, *V.sinocinerea*, and *V.bellula* in its glabrous to hirtellously pubescent abaxial mature leaf surface (vs. abaxially densely arachnoid tomentose in *V.bryoniifolia*, *V.sinocinerea*, and *V.bellula*). It differs from *Vitisflexuosa* in its leaves varying from unlobed to 3–7 lobed (vs. unlobed to slightly 3-lobed leaves in *V.flexuosa*), tendrils unbranched or bifurcate from upper half (vs. tendrils bifurcate from approximately midway in *V.flexuosa*), lack of arachnoid tomentum (vs. with sparse arachnoid tomentum to glabrescent in *V.flexuosa*), and subcordate to cordate or sometimes truncate leaf base (vs. subtruncate or slightly subcordate leaf base in *V.flexuosa*).

#### Description.

Woody climber, sprawling to moderately high climbing, sparsely branched. Branchlets terete, glabrous, with longitudinal ridges, tendrils unbranched or bifurcate from upper half. Leaves simple; stipules ovate-elliptic or lanceolate, 1–4 mm; petiole 2–6 cm, hirtellous or glabrous; blade 3–10 × 3–9 cm, unlobed to slightly 3-lobed, or 3–7 lobed, apex acute to acuminate, base subtruncate or subcordate to cordate, abaxial surface usually glabrous, veins and vein axils hirtellous, adaxial surface glabrous, basal veins 5, with lateral veins 4–6 pairs. veinlets inconspicuous, base subcordate to cordate, occasionally truncate. Margin with 8–16 obtuse teeth on each side. Inflorescences a panicle, 3.4–9 cm, leaf-opposed, peduncle 1–6.4 cm, pedicel 1–2.5 mm, usually glabrous. Calyx shallow and saucer-shaped, glabrous. Petals 5, occasionally 6, connate distally, forming calyptra. Berries black, globose, 5–8 mm in diam. Seeds obovoid or obovoid-elliptic, 3–4 × 2–3 mm, abaxial surface with a round to elliptic chalaza, adaxial surface with 2 furrows (ventral infolds) running ½ through seed length, endosperm M-shaped in transverse section.

#### Additional specimens examined.

China. Hubei. Tianmen City, Mawan Town, 15 Jul 2020, fr, *X.Q. Liu 944* (CCAU); Wuhan, Shizhishan, 26 m, May 6, 2021, in flower, *X. Q. Liu 155* (CCAU) (see photos in Suppl. material 1: Fig. S1, Suppl. material 2: Fig. S2, Suppl. material 3: Fig. S3).

#### Phenology.

Flowering from March to May, fruiting from July to October.

#### Etymology.

The specific epithet is derived from the type locality, Shizishan, Wuhan, Hubei, China. The Chinese name is given as “狮子山葡萄”.

#### Distribution and habitat.

**Figure 7.**
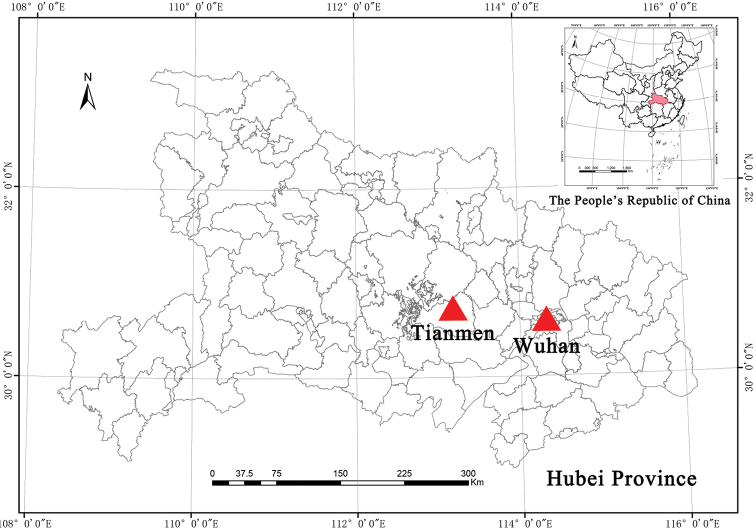
Distribution of *Vitisshizishanensis* sp. nov. (triangle).

The new species is currently known from Wuhan and Tianmen in Hubei province, China (Fig. 7). It occurs on the scrubland and the roadside of farmland at an altitude of ca. 10–50 m.

*Vitisshizishanensis* is morphologically similar to *V.bryoniifolia*, *V.flexuosa*, *V.sinocinerea*, and *V.bellula.* Detailed morphological comparisons among the three species are provided in Table 1. These characters were based on field observations, and herbarium and literature studies (Li et al. 1996; Chen et al. 2007; Wan et al. 2008).

**Table 1.** Morphological comparisons among *Vitisshizishanensis*, *V.bryoniifolia*, *V.flexuosa*, *V.sinocinerea*, and *V.bellula*.

Characters	*V.shizishanensis*	*V.flexuosa*	*V.bryoniifolia*	*V.sinocinerea*	*V.bellula*
tendrils	unbranched or bifurcate in the upper half	bifurcate to the middle	bifurcate	unbranched or bifurcate	unbranched or bifurcate
Size of leaves	ca. 3–10 × 3–9 cm	ca. 5–12 × 3.5–10 cm	ca. 2.5–8 × 2–5 cm	ca. 3–8 × 3–6 cm	ca. 3–7 × 2–4 cm
Leaf base	subtruncate or subcordate to deeply cordate	slightly subcordate or subtruncate, rarely cordate	cordate or deeply cordate	subcordate or subtruncate	subcordate, subtruncate, or subrounded
Shape of leaves	unlobed to 3–7 lobed	unlobed to slightly 3-lobed	unlobed to 3–7 lobed	3-lobed or inconspicuously divided	unlobed
Abaxial mature leaf surfaces	usually glabrous	with sparse arachnoid tomentum to glabrescent	with dense arachnoid tomentum	with dense arachnoid tomentum	with dense arachnoid tomentum
Size of fruits	5–8 mm in diam	8–10 mm in diam	5–8 mm in diam	6–10 mm in diam	6–10 mm in diam
Altitude	10–50 m	100–2300 m	100–2500 m	200–2800 m	400–1600 m
Distribution	China (Hubei)	China, India, Japan, Laos, Nepal, Philippines, Thailand, Vietnam	China (Anhui, Fujian, Guangdong, Guangxi, Hebei, Hubei, Hunan, Jiangsu, Jiangxi, Shaanxi, Shandong, Shanxi, Sichuan, Yunnan)	China (Fujian, Hubei, Hunan, Jiangsu, Jiangxi, Taiwan, Yunnan, Zhejiang)	China (Guangdong, Guangxi, Hubei, Hunan, Sichuan)

## Supplementary Material

XML Treatment for Vitis shizishanensis

## Supplementary materials

Supplementary material 1 Figure S1 Data type Jpg file. Explanation note Isotype of *Vitisshizishanensis* Z.Y.Ma, J. Wen, Q. Fu & X-Q. Liu (X.Q. LIU 155) with 5–7 lobed leaves. https://binary.pensoft.net/file/604897This dataset is made available under the Open Database License (http://opendatacommons.org/licenses/odbl/1.0/). The Open Database License (ODbL) is a license agreement intended to allow users to freely share, modify, and use this Dataset while maintaining this same freedom for others, provided that the original source and author(s) are credited.Zhi-Yao Ma, Jun Wen, Qiang Fu, Xiu-Qun Liu

Supplementary material 2 Figure S2 Data type Jpg file. Explanation note Isotype of *Vitisshizishanensis* Z.Y.Ma, J. Wen, Q. Fu & X-Q. Liu (X.Q. LIU 155) with male flowers. https://binary.pensoft.net/file/604898This dataset is made available under the Open Database License (http://opendatacommons.org/licenses/odbl/1.0/). The Open Database License (ODbL) is a license agreement intended to allow users to freely share, modify, and use this Dataset while maintaining this same freedom for others, provided that the original source and author(s) are credited.Zhi-Yao Ma, Jun Wen, Qiang Fu, Xiu-Qun Liu

Supplementary material 3 Figure S3 Data type Jpg file. Explanation note Isotype of *Vitisshizishanensis* Z.Y.Ma, J. Wen, Q. Fu & X-Q. Liu (X.Q. LIU 944) with fruits. https://binary.pensoft.net/file/604899This dataset is made available under the Open Database License (http://opendatacommons.org/licenses/odbl/1.0/). The Open Database License (ODbL) is a license agreement intended to allow users to freely share, modify, and use this Dataset while maintaining this same freedom for others, provided that the original source and author(s) are credited.Zhi-Yao Ma, Jun Wen, Qiang Fu, Xiu-Qun Liu
