# Supplementary figures and images for: Vitisshizishanensis, a new species of the grape genus from Hubei province, China
Source: PhytoKeys. 2021 Nov 2;184:45–54. doi: 10.3897/phytokeys.184.70045 (PMC8578147; doi:10.3897/phytokeys.184.70045)

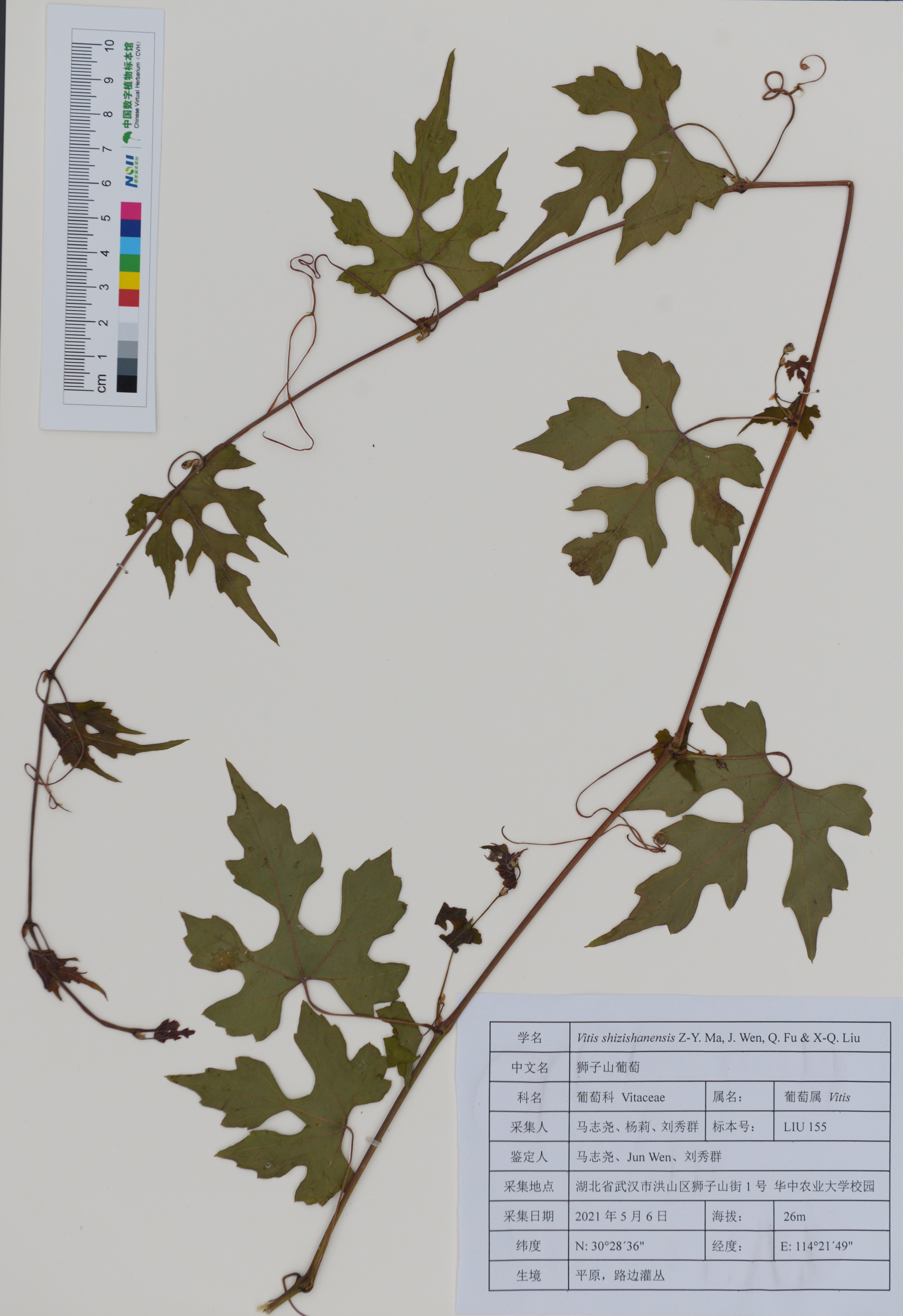

Supplement: Supplementary material 1 — Figure S1 [file phytokeys-184-045-s001.jpg]

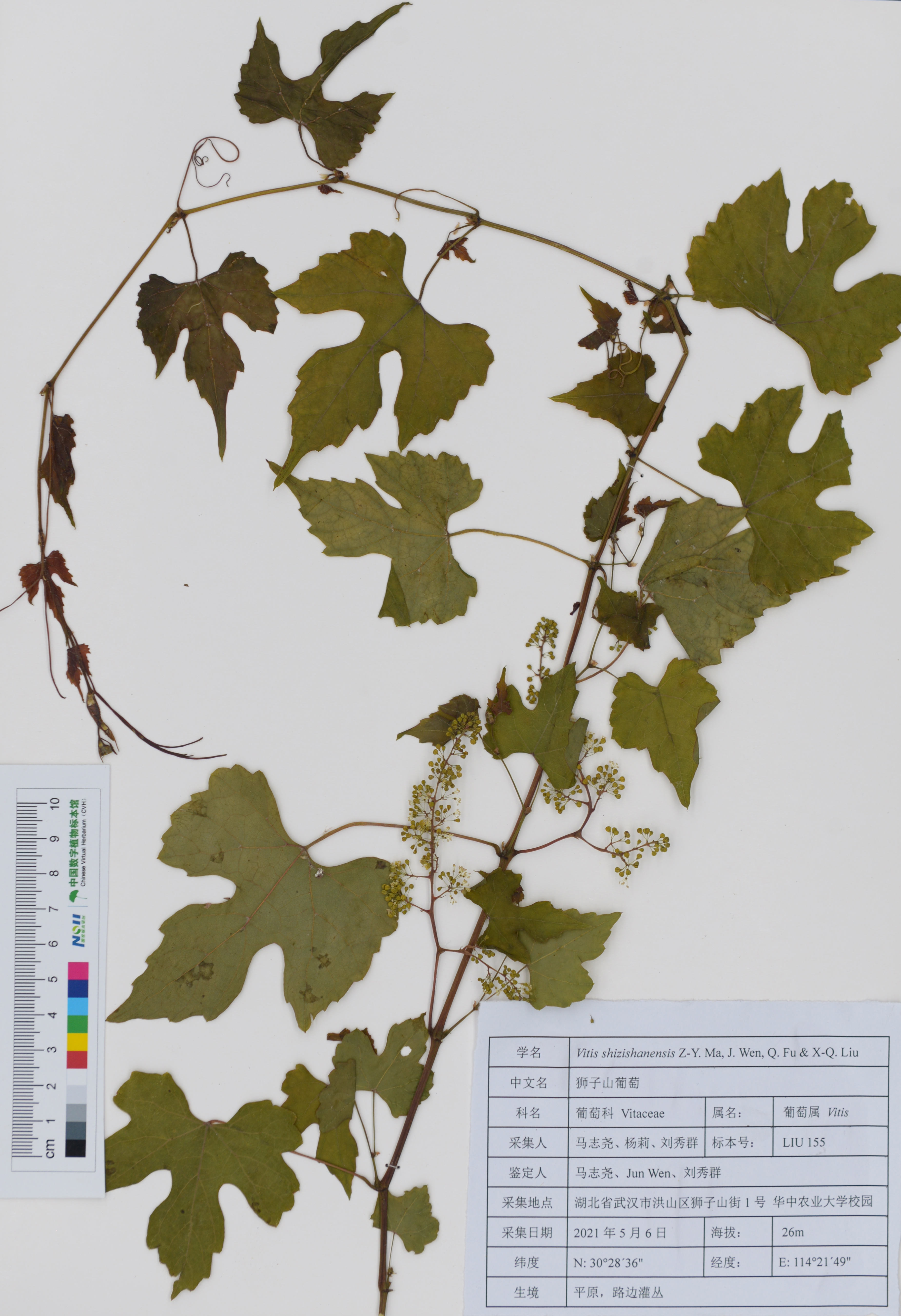

Supplement: Supplementary material 2 — Figure S2 [file phytokeys-184-045-s002.jpg]

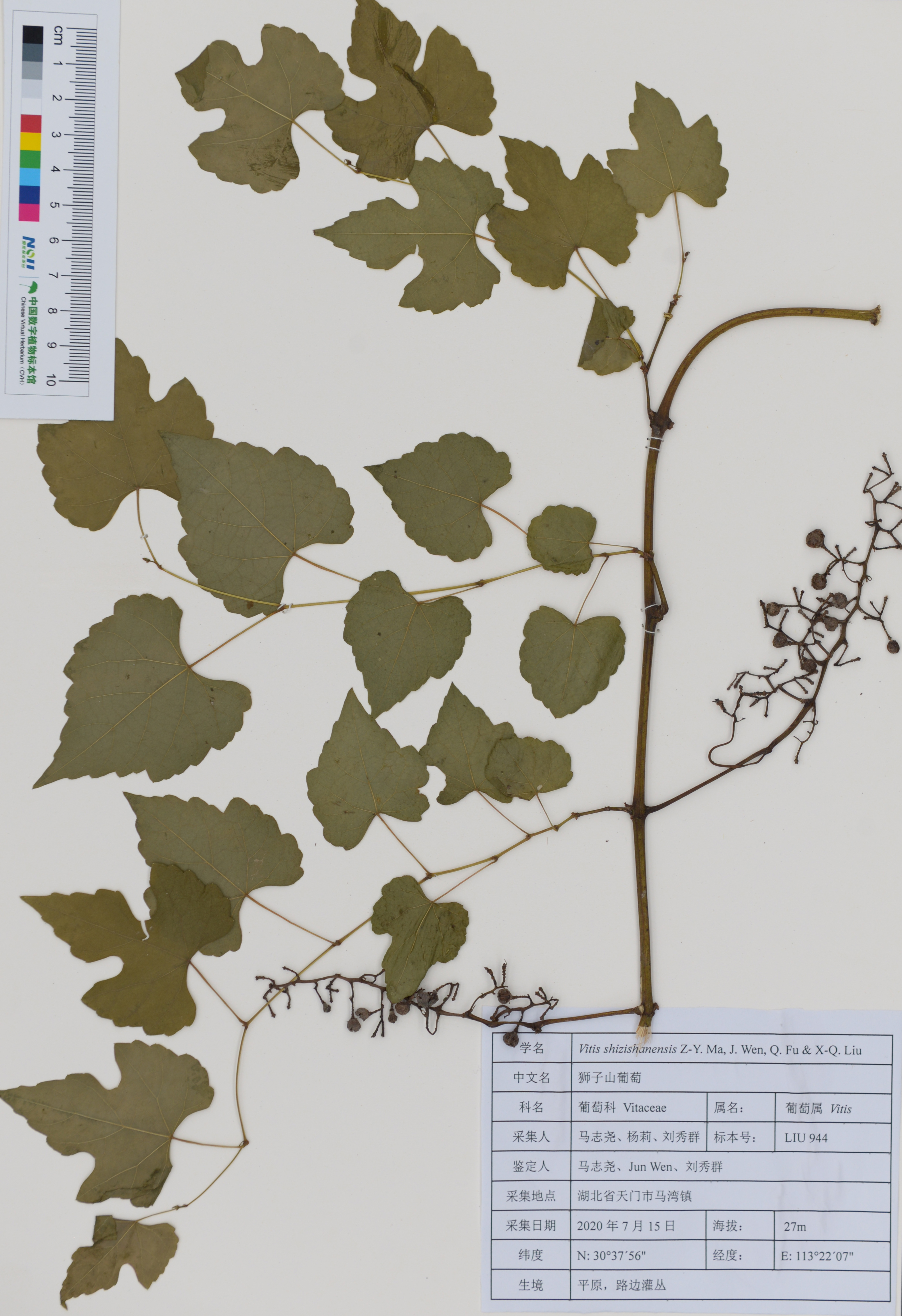

Supplement: Supplementary material 3 — Figure S3 [file phytokeys-184-045-s003.jpg]
